# 

**Published:** 1896-02-29

**Authors:** 


CADBURY'S COCOA

"The standard of highest purity at present attainable."—*Lancet.*

CADBURY'S COCOA

NO ALKALIES USED.

### THE HOSPITAL

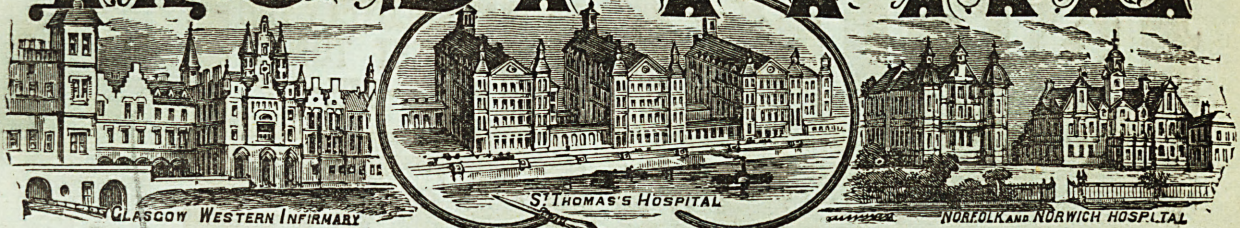

No. 492. Vol. XIX.

SATURDAY, FEB. 29TH, 1896.

WITH EXTRA NURSING SUPPLEMENT.

[Registered at the General Post Office as a Newspaper for transmission in the United Kingdom and Abroad.]

PRICE TWOPENCE.

Per Annum, 10s. 6d., post free.

Monthly Parts, 6d.; post free, 8d.

Ditto per Annum, 8s.6d., post free.

